# Subregional origins of emerging SARS-CoV-2 variants during the second pandemic wave in Côte d’Ivoire

**DOI:** 10.1007/s11262-023-01984-2

**Published:** 2023-03-18

**Authors:** Etilé A. Anoh, Oby Wayoro, Pacôme Monemo, Essia Belarbi, Andreas Sachse, Eduan Wilkinson, James E. San, Fabian H. Leendertz, Bamourou Diané, Sébastien Calvignac-Spencer, Chantal Akoua-Koffi, Grit Schubert

**Affiliations:** 1 Centre Hospitalier et Universitaire de Bouaké, Bouaké, Côte d’Ivoire; 2 Université Alassane Ouattara de Bouaké, Bouaké, Côte d’Ivoire; 3 Robert Koch Institute, Nordufer 20, 13353 Berlin, Germany; 4 Helmholtz Institute for One Health, Greifswald, Germany; 5 KwaZulu-Natal Research Innovation and Sequencing Platform (KRISP), Nelson R Mandela School of Medicine, University of KwaZulu-Natal, Durban, South Africa; 6 Centre for Epidemic Response and Innovation (CERI), School for Data Science and Computational Thinking, Stellenbosch University, Stellenbosch, South Africa

**Keywords:** SARS-CoV-2, VOC, VOI, Whole-genome sequencing, Phylogeography, Sub-Saharan Africa

## Abstract

Severe acute respiratory syndrome coronavirus 2 (SARS-CoV-2) variants with increased transmissibility, virulence and immune escape abilities have heavily altered the COVID-19 pandemic’s course. Deciphering local and global transmission patterns of those variants is thus key in building a profound understanding of the virus’ spread around the globe. In the present study, we investigate SARS-CoV-2 variant epidemiology in Côte d’Ivoire, Western sub-Saharan Africa. We therefore generated 234 full SARS-CoV-2 genomes stemming from Central and Northern Côte d’Ivoire. Covering the first and second pandemic wave the country had been facing, we identified 20 viral lineages and showed that in Côte d’Ivoire the second pandemic wave in 2021 was driven by the spread of the Alpha (B.1.1.7) and Eta (B.1.525) variant. Our analyses are consistent with a limited number of international introductions of Alpha and Eta into Côte d’Ivoire, and those introduction events mostly stemmed from within the West African subregion. This suggests that subregional travel to Côte d’Ivoire had more impact on local pandemic waves than direct intercontinental travel.

## Introduction

The coronavirus disease 2019 (COVID-19) pandemic, caused by the severe acute respiratory syndrome coronavirus 2 (SARS-CoV-2), is driven by the emergence and spread of virus variants with clinically relevant mutations that lead to increased transmissibility, virulence and immune escape abilities [1, 2]. Deciphering local and global transmission patterns of those variants is thus key in building a profound understanding of the pandemic’s spread and will inform public health policy. While genomic surveillance was deployed on a global scale, SARS-CoV-2 genomic data have accumulated relatively slower in West sub-Saharan Africa (West Africa, [3]).

The virus has spread on the entire African continent in multiple pandemic waves. The third and fourth pandemic waves were essentially driven by the uniform expansion of Delta and Omicron, but the first and second waves were respectively coined by the co-circulation of multiple SARS-CoV-2 lineages (mostly PANGO B.1 viruses) and the Alpha (B.1.1.7) and Beta (B.1.351) variants [3, 4]. The second wave was also characterized by the more limited spread of several local variants of interest (VOI) with genetic changes predicted or known to affect virus characteristics, such as Eta (B.1.525, [5]) and A.27 [6].

A country where comprehensive investigation of variant epidemiology at a national scale is still lacking, is Côte d’Ivoire in the Southern coastal region of West Africa. By September 2022, Côte d’Ivoire had observed 86,821 cases of COVID-19, with 819 deaths recorded (https://www.worldometers.info). Here, we set out to shed light on the evolution and spread of SARS-CoV-2 and its VOC/VOI in Côte d’Ivoire during the first and second pandemic waves, by sequencing viral genomes and subsequently using them for in-depth phylogeographic analyses.

## Materials and methods

### SARS-CoV-2 whole-genome sequencing and genome assembly

We obtained nucleic acids extracted from naso/oropharyngeal specimens from the national SARS-CoV-2 surveillance program of Côte d'Ivoire. Those samples had been tested positive for SARS-CoV-2 by real-time PCR. Viral RNA extracted from SARS-CoV-2 positive respiratory specimen was transcribed into cDNA using the SuperScript™ IV First Strand Synthesis Kit (Invitrogen), following manufacturer’s instructions. Tiled amplicons of each about 400 bp in length were generated by two multiplex PCRs, using primer scheme V3 and following reaction and cycling conditions of the ARTIC protocol [7]. The two amplicon sets were pooled, and sequencing libraries were prepared according to [7], using the NEBNext® Companion Module for Oxford Nanopore Technologies® (ONT) Ligation Sequencing, and the ONT Native Barcoding Expansion Kit 1–96 kit for multiplexing samples. Up to 48 pooled libraries, including one negative control, were sequenced on an ONT MinION, using R9.4.1 flow cells.

Bases were called with the MinKNOW software, while we followed the ARTIC bioinformatics protocol [8] for demultiplexing (with Guppy 4.2.2., requiring barcodes at both ends of reads), read filtering, primer trimming, variant calling, mapping to reference genome Wuhan-Hu-1 (GenBank Accession: MN908947.3) and consensus sequence building. Rare single nucleotide polymorphisms and deletions in the assembled genomes were manually inspected in Geneious Prime® 2021.2.2 and ambiguous positions marked as N. We next excluded sequences identified as being of low quality by NextClade (https://clades.nextstrain.org), those with missing sampling dates, those with < 90% coverage, those with > 40 SNPs, those with > 10 ambiguous base-calls per genome, and those with clustered SNPs. Of the 461 specimen we had attempted to sequence, 234 high quality complete or near-complete SARS-CoV-2 genomes were retrieved and deposited on GISAID, the Global Initiative on Sharing All Influenza Data [9].

### SARS-CoV-2 linneage assignment

We assigned the Côte d'Ivoire SARS-CoV-2 genomes to virus lineages defined in the dynamic nomenclature of SARS-CoV-2 lineages (pango-nomeclature, [10]) via pangolin v1.2.105, with pangoLEARN version from 26th December 2021. Variants of concern (VOC) and variants of interest (VOI) as of May 31st 2021 were labeled based on the naming system by the World Health Organization for key SARS-CoV-2 variants as of May 31st 2021 [11]. Namely, pango-lineage B.1.1.7 was designated the Alpha variant, pango-lineage B.1.351 the Beta variant, and pango-lineage B.1.525 the Eta variant. Variant dynamics over time were visualized in R using the Treemap package [12].

### Phylogeographic reconstruction

We retrieved respective sequence data sets compiled by Emma Hodcroft and Richard Neher (Neherlab) for Alpha (4886 sequences) and Eta (4965 sequences) variants from Nextstrain [13] on September 30th 2021, and merged each data set with high quality sequences generated from Côte d’Ivoire for variant Alpha (33 sequences) and Eta (45 sequences), respectively. We then restricted both datasets to sequences sampled prior to June 1st 2021 to reflect the sampling period in Côte d’Ivoire, and followed the same criteria for retaining only sequences of high quality for phylogenetic analyses described above and used in [14]. Final Alpha and Eta datasets included 3662 and 4454 complete or near complete high-quality sequences, respectively. Both downloaded sequence data sets were aligned against the Côte d'Ivoire genomes with MAFFT v7.471 [15]. The first 100 and last 50 bases and positions 13,402, 24,389 and 24,390, relative to reference strain sequence Wuhan-Hu-1 (Accession Number NC_045512) were masked to avoid ambiguities through primer contamination. Maximum likelihood trees for each of the alignments were inferred in IQ-TREE multicore version 2.1.4-beta [16], using IQ-TREE’s ModelFinder for identifying best fitting rate variation models [17]. We performed 100 bootstrap replicates also in IQ-TREE to get some measure of confidence of phylogenetic tree branches, and to feed into sensitivity analyses for transmission of viral strains across geographic locations (see below). Alpha and Eta trees were inferred with a General time reversible (GTR) model of nucleotide substitution, using empirical base frequencies (+F), a proportion of invariable sites (+I) and a discrete Gamma model with default 4 rate categories (G4).

We next produced a time scaled phylogenetic tree based on sampling dates, using a fixed rate of 8.0 × 10^–4^ nucleotide substitutions per site per year, with a standard deviation of 4.0 × 10^–4^, in TreeTime v0.8.6 [18]. Prior to final tree building, outliers that deviated more than three interquartile ranges from the root-to-tip regression were removed.

### Introduction analysis

The dated phylogenetic tree was used to fit a mugration model, which treats locations (in our case countries) as discrete traits that evolve through the phylogeny. Mapping countries to tips and internal nodes of the tree allows to estimate the number of viral transmission events for the Alpha and Eta lineage between Côte d’Ivoire and the rest of the world, which was done via a Python script developed by the authors (E. Wilkinson, J.E. San). We performed a sensitivity test to examine the robustness of this introduction analysis towards which time-scaled phylogenetic tree is used as starting point for subsequent analyses. For this, we replicated the inference of a maximum likelihood phylogenetic tree in IQ-TREE ten times, starting with different seeds and re-ran the entire workflow from each tree to reconstruct ancestral states and infer introduction events. We plotted average number of introductions into Côte d’Ivoire with standard errors over time. Plots and phylogenetic trees were visualized using R ggplot2 [19].

## Results and discussion

### SARS-CoV-2 variant distribution

Between May 23rd 2020 and May 31th 2021, 4071 naso/oropharyngeal specimens from COVID-19 suspect cases had been received by the Centre Hospitalier et Universitaire (CHU) de Bouaké, of which 719 specimens tested positive for SARS-CoV-2. An additional 8 SARS-CoV-2 positive nucleic acids were obtained from a running surveillance study on acute respiratory infections in the Bouaké region and Western Côte d’Ivoire (N_tested_ = 828, [20]). We generated 234 high quality SARS-CoV-2 genome sequences from Côte d’Ivoire following the ARTIC protocol for nanopore sequencing and assigned those to 20 viral lineages. Most genomes stemmed from districts in Central Côte d’Ivoire (Lacs, Haut-Sassandra, Marahoué, Gbèke, Worodougou-Bere), while few genomes represented the Northern (Savanes) and Western (Montagnes) parts of the country. All laboratory activities were carried out at the CHU de Bouaké, building local capacity for genomic surveillance.

**Fig. 1:**
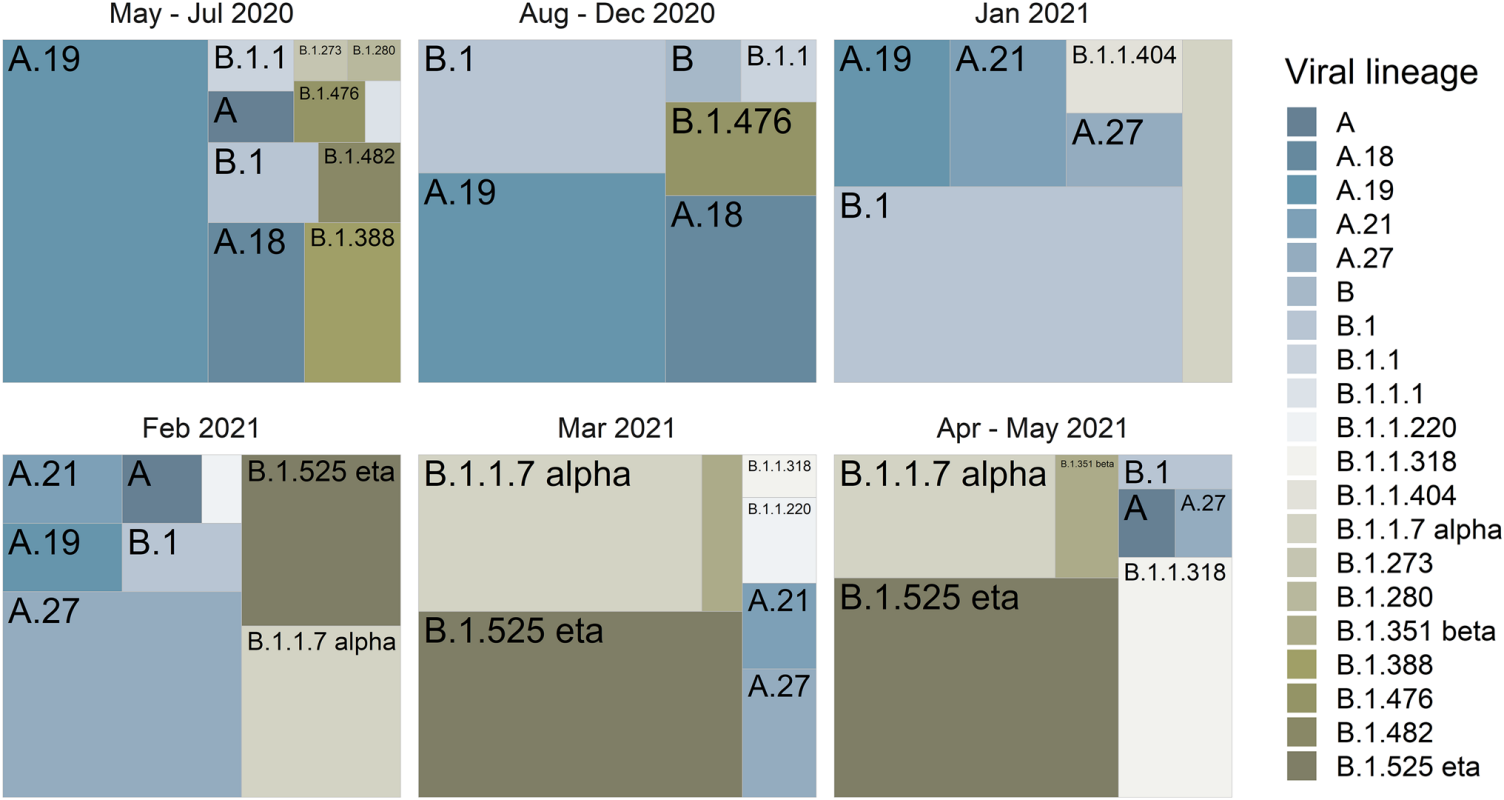
Distribution of severe acute respiratory syndrome coronavirus 2 (SARS-CoV-2) viral lineages among 234 genomes sequenced at the Centre Hospitalier et Universitaire de Bouaké in Côte d’Ivoire. Data were subdivided into six sample collection periods, aiming to balance sampling effort: May–July 2020 (*N*  = 62), August–December 2020 (*N*  = 29), January 2021 (N = 16), February 2021 (*N*  = 50), March 2021 (*N* = 43), April–May 2021 (*N*  = 34). Viral lineage assignment was conducted using pangolin (version 2.3.8, April 2nd 2021), and the plot was drawn with R, package Treemap.

The first pandemic wave within Côte d’Ivoire lasted from June to August 2020, and the second from late December 2020 to April 2021. A—lineages (A.19, A.18) dominated through to December 2020 (Fig. 1), which were also typical for other West African countries at that time, but not in East and Southern Africa (e.g., [21, 22]). In January 2021, the picture changed rapidly when VOC and VOI started to outgrow previous lineages (Fig. 1). We detected 2 VOC — Alpha (first detection January 15th 2021) and Beta (first detection March 6th 2021) — as well as VOI Eta (B.1.525, first detection February 8th 2021), A.27 (first detection January 19th 2021) and B.1.1.318 (March 26th 2021). The frequency of VOC/VOI steadily increased to make up 77.9% of sequenced genomes in May 2021 (Fig. 1).

### Variant circulation during the second pandemic wave

VOI A.27 had a brief high, representing one third of all genomes (36%) in February 2021 (Fig. 1). As a number of VOC/VOI do, A.27 genomes harbor several lineage defining mutations, some of which are potentially linked to increased transmissibility or immune escape [6]. A.27 most likely emerged in West Africa, from which it spread to 31 countries ([6], Table 1). However, after this initial burst, A.27 was quickly superseded by apparently more easily propagated VOC/VOI. In line with previous findings from West Africa [23], VOI B.1.1.318 as well as VOC Beta circulated at only low frequencies in Côte d’Ivoire overall (Fig. 1, Table 1).

**Table 1:** Variants of concern (VOC) and variants of interest (VOI) circulating in Côte d’Ivoire during the second wave of COVID-19 in early 2021, and their occurrence in other West African countries

SARS-CoV-2 variant	Sequences worldwide (GISAID*, 08.09.2022)	% (N) West African sequences of worldwide data (GISAID*, 08.09.2022)	West sub-Saharan African countries reporting the variant
A.27	874	28% (245)	Benin, Burkina Faso, Côte d’Ivoire, Gambia, Ghana, Guinea, Nigeria, Senegal, Togo
B.1.1.318	4090	18.5% (757)	Benin, Burkina Faso, Côte d’Ivoire, Gambia, Ghana, Guinea, Liberia, Nigeria, Senegal, Togo
B.1.1.7-Alpha	1,192,869	0.12% (1394)	Benin, Burkina Faso, Côte d’Ivoire, Gambia, Ghana, Guinea, Guinea-Bissau, Liberia, Mali, Nigeria, Senegal, Togo
B.1.351-Beta	45,444	0.2% (72)	Benin, Côte d’Ivoire, Ghana, Guinea-Bissau, Liberia, Nigeria, Senegal, Togo
B.1.525-Eta	10,223	20% (2042)	Benin, Burkina Faso, Côte d’Ivoire, Gambia, Ghana, Guinea, Guinea-Bissau, Liberia, Mali, Nigeria, Senegal, Togo

*GISAID, Global Initiative on Sharing All Influenza Data

Indeed, VOC Alpha and VOI Eta quickly rose to being the most prevalent variants in Côte d’Ivoire during the second pandemic wave the country was facing. From January 2021 on, both variants increased in frequency through to May 2021, right after the peak of A.27, and presented overall 22.9% and 31.3% of all genomes sampled since January 2021, respectively. Alpha is characterized by 21 lineage defining mutations or deletions, including eight changes within the viral Spike gene which are linked to increased ACE-2 receptor binding affinity and innate and adaptive immune evasion [24]. The variant was the dominating VOC in West Africa at the onset of the second pandemic wave ([5, 25–28], Table 1). Subsequently, it was outgrown by Eta in most of West Africa, but not in Côte d’Ivoire where Alpha remained most frequently found.

Unlike Alpha, which was originally introduced into West Africa multiple times from mainly Europe [27], Eta likely emerged in Nigeria in November 2020 [3] and had propagated via sustained regional transmission among neighboring countries to become frequent in West Africa by February/March 2021 ( [3, 5, 25, 26, 28, 29], Table 1). Eta exhibits mutations in the Spike protein that facilitate enhanced viral entry and decrease the effectiveness of neutralizing antibodies [5]. Of note, Eta persisted in the region even after the introduction of a rather rare lineage of the highly virulent Delta VOC [5]. Whether the same happened in Côte d’Ivoire remains to be investigated by continued sequencing efforts.

### Origins of the Alpha and Eta variant circulating in Côte d’Ivoire

**Fig. 2:**
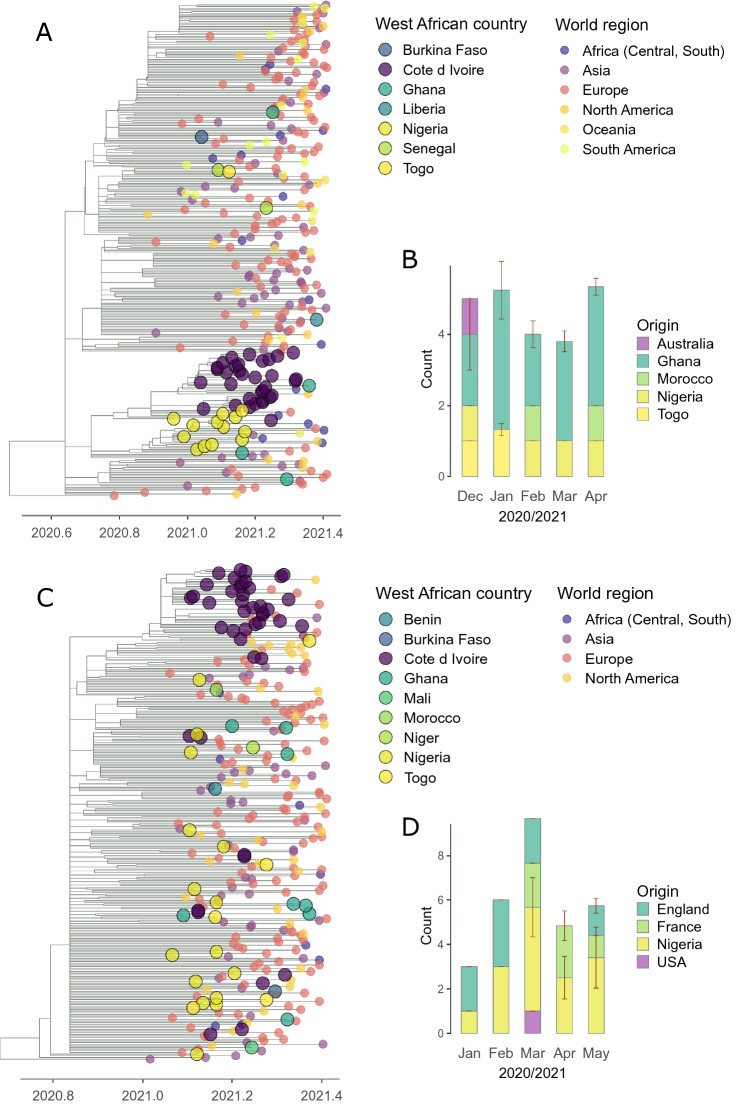
**A**, **C** Subsampled time-scaled phylogeny of severe acute respiratory syndrome coronavirus 2 (SARS-CoV-2) lineage Alpha (**A**) and Eta (**C**). 300 sequences were subsampled from a global dataset as to maximize genetic distances, while retaining all genomes from Côte d’Ivoire. The branches are scaled in decimal time, and sampling dates are capped at May 31st 2021 (decimal date 2021.41), the latest sampling month in Côte d’Ivoire in this study. Sequences originating from West Africa are indicated by large circles. **B**, **D** Number of importation events of severe acute respiratory syndrome coronavirus 2 (SARS-CoV-2) lineage Alpha (**B)** and Eta **(D)** into Côte d’Ivoire. Sampling dates are capped at May 31st 2021, the latest sampling month in Côte d’Ivoire in this study. Standard error bars are derived from replicating the introduction analysis ten times starting from different maximum likelihood phylogenetic trees.

In order to shed light on the origins of Alpha and Eta circulation in Côte d’Ivoire, we generated time scaled phylogenies (Fig 2A and C) and applied a “mugration” model to estimate introduction rates and origins for each variant, using publicly available variant-specific datasets and all Ivorian sequences generated in this study between May 2020 to May 2021 (Alpha dataset: 3662 genomes including 33 from Côte d’Ivoire; Eta dataset: 4454 genomes including 45 from Côte d’Ivoire). For Alpha, we inferred an average of 15 introductions into Côte d’Ivoire between December 2020 and April 2021 (range = 14–17), with virtually all (15; range = 14–15) originating from West Africa (Fig. 2B). For Eta, we estimated an average of 26 introductions from January to May 2021 (range = 25–26; Fig. 2D). Nigeria, the country where Eta likely emerged, appeared as the most frequent source of introductions into Côte d’Ivoire (17 introductions; range = 12–19), pointing again at primarily subregional spread of this variant into Côte d’Ivoire. Yet, particularly England was also an (apparent) important contributor to Eta entry into Côte d’Ivoire. This observation might however reflect genome sampling biases, as genomic surveillance was much more intense in England than in Nigeria (Eta genomes produced from English cases: 227,318; from Nigerian cases: 264; in our dataset: 338 from England, 254 from Nigeria).

## Conclusions

Taken together, our analyses are consistent with a limited number of international introductions of Alpha and Eta into Côte d’Ivoire. Importantly, these introduction events mostly stemmed from within the West African subregion and this, irrespective of the origin of the variant, suggests that subregional travel to Côte d’Ivoire had more impact on local pandemic waves than direct intercontinental travel to the country. The subsequent rapid propagation of both variants within Côte d’Ivoire seeded the second wave of the pandemic and might have been facilitated by founder effects at a time when case numbers had dropped significantly. An important limitation of our study is it being geographically limited to Central and Northern Côte d’Ivoire, while further investigations in the coastal region, where Abidjan acts as the country’s hub for intercontinental travel, will be needed to fully understand SARS-CoV-2 dynamics within Côte d’Ivoire.

Monitoring the spread and possibly local emergence of virus variants provides information guiding governmental measures towards pandemic control. Hence, reinforcing genomic surveillance on the African continent remains an important regional and global task.

## Data Availability

SARS-CoV-2 consensus genome sequences used in this study were uploaded to the Global Initiative on Sharing All Influenza Data (GISAID) portal.
